# Real-time particle size analysis using focused beam reflectance measurement as a process analytical technology tool for continuous microencapsulation process

**DOI:** 10.1038/s41598-021-98984-9

**Published:** 2021-09-29

**Authors:** Muhaimin Muhaimin, Anis Yohana Chaerunisaa, Roland Bodmeier

**Affiliations:** 1 Faculty of Pharmacy, Universitas Padjadjaran, Jl. Raya Jatinangor Km 21, Sumedang, Jawa Barat, 45363 Indonesia; 2 College of Pharmacy, Freie Universität Berlin, Kelchstr. 31, Berlin, 12169 Germany

**Keywords:** Chemistry, Materials science

## Abstract

The online real-time particle size analysis of the microencapsules manufacturing process using the continuous solvent evaporation method was performed using focused beam reflectance measurement (FBRM). In this paper, we use FBRM measurements to investigate the effects of polymer type and compare the size distributions to those obtained using other sizing methods such as optical microscope and laser diffraction. FBRM was also utilized to measure the length-weighted chord length distribution (CLD) and particle size distribution (PSD) online during particle solidification, which could not be done with laser diffraction or nested sieve analysis. The chord lengths and CLD data were taken at specific times using an online FBRM probe mounted below the microparticle. The timing of the FBRM determinations was coordinated with the selection of microparticle samples for particle size analysis by optical microscope and laser diffraction calculation as a reference. For all three produced batches tested, FBRM, laser diffraction, and sieve analysis yielded similar results. Hardening time for the transformation of emulsion droplets into solid microparticles occurred within the first 10.5, 19, 25, 30, and 55 min, according to FBRM results. The FBRM CLDs revealed that a larger particle size mean resulted in a longer CLD and a lower peak of particle number. The FBRM data revealed that the polymer type had a significant impact on microparticle CLD and the transformation process.

## Introduction

Microencapsulation is a typical unit operation used in the production of solid dosage types including tablets, capsules, and sachets. Microparticle friability, microparticle flowability, tablet weight variance, tabletability, microparticle bulk density, tablet porosity, and tablet dissolution rate are all important parameters to be considered^1–5^. The requirement to ensure the manufacture of a repeatable product necessitates the ability to produce microparticulate particle sizes and distributions within defined limits^1–5^.

Solvent evaporation method is a popular technique for the encapsulation of drugs within polymeric microparticles. Water insoluble polymers are usually used as encapsulation matrix for these microparticles. Microparticles have been prepared with a wide range of polymers and polymer blends^1,2^. Various types of polymer with different physical properties (such as biodegradable, non-biodegradable, permeable, etc.) have been used in microparticles preparation. They are poly(є-caprolactone), poly(lactic-co-glycolic acid), copolymers of ethyl acrylate, and ethyl cellulose^5–8,10–12^. Information about the microparticle hardening rate which is an important factor in determining particle size from these polymers is not available. The solidification rate of polymeric microparticles is also an important parameter influencing the particle size, the encapsulation efficiency and the initial burst in microparticulate systems. According to Mehta et al., solubilities of polymers in organic solvents determine the solidification rate of the polymers during the microparticle preparation process, which in turn affects microparticle properties such as particle size^13^. Thus, it is necessary to know the effect of polymer properties on the polymeric microparticle hardening time as well as solidification rate.

When using solvent evaporation techniques to prepare microparticles, the hardening speed of microparticles is a crucial factor that significantly affects drug release^3^. Slow hardening of droplets or emulsions causes drug compound diffusion, resulting in poor encapsulation content^1,3,12–15^. The hardening speed of microparticles in the solvent evaporation cycle is affected by the solubility of the polymer in organic solvents, which impact on microparticle properties such as particle size, volume of encapsulated drugs, matrix porosity, solvent residues, and initial drug release^16–19^. Polymers with various physical properties (such as solubility, molecular weight, reactivity, viscosity, biodegradability, permeability, and so on) have been applied to create microparticles^19–22^.

Laser diffraction and sieve analysis are commonly used techniques to measure granule particle size post-milling. Sieve analysis equipment is relatively inexpensive, and it is still widely used at line and for quality control purposes. Although sieve analysis is the most common method for granule particle size analysis in the pharmaceutical industry, the analysis is time consuming and difficult to perform for oily or cohesive powders or granules with particle sizes of less than 25 μm. If the particles retained on any sieve and found to be aggregates, the method is not easily reproducible. Accordingly, dilution and sampling are used in these processes, which can result in changes in the droplet/particle size distribution due to break-up/coagulation or coalescence. Sampling techniques are often non-representative and can be used with caution. The Focused Beam Reflectance Method (FBRM) technique allows for in-situ measurements to monitor particle/droplet size in real time during the microencapsulation process^23–26^. Lasentec invented the FBRM process, which can quantify particle size in the range of 0.1–1000 μm. This instrument provides data from on-line and real-time measurements, allowing particle size data and suspension population patterns to be observed. It's been largely applied to monitor crystallization processes. This device can also visually track the transformation of emulsion droplets into stable microparticles, known as hardening time.

Microparticle particle size is measured using laser diffraction and sieve analysis during the solvent evaporation process. Sieve measurement equipment is cheap, and it is still commonly applied on the internet and for quality monitoring. While sieve analysis is the most common tool for microparticulate particle size analysis in the pharmaceutical industry, it is time consuming and difficult to conduct for oily or cohesive powders, microparticulate, or granules with particle sizes less than 25 µm. The process is not readily repeatable if the particles retained on some sieve are aggregates rather than single particles. The second biggest particle dimension, as determined by analytical sieving, is affected by particle structure^26–32^.

Laser diffraction (LD) can be used as an in-process method or as an off-line method^33^. A dispersed sample passes through a beam of monochromatic light causing light scattering, which is measured as a function of scattering angle by a multi-element detector. In comparison to an analytical sieve apparatus, laser diffraction instrumentation is still comparatively costly. Inline real-time particle size estimation and input control is an ideal method of microparticulate size analysis because it reduces human error, reduces analytical time and expense, reduces manufacturing cycle time, increases material throughput, and improves microparticulate size control^29–32^. Recent LD particle size analyzers are based on Mie’s theory^33,34^. It predicts the scattering intensity induced by particles, irrespective of the fact whether they are transparent or opaque. LD particle size analyzers that use Mie’s theory (e.g., Mastersizer) base their particle size calculation on the assumption that particles are spherical, which is rarely true. This is a solution to deal with the fact that the only shape that can be described by a single dimension is the sphere. LD results are generally presented as a volume-weighted particle size distribution.

To date very little work has been carried out on the online monitoring of microparticles during their formation. In a solvent evaporation process, solidification of the emulsion droplets and particle size changes occur after emulsifying the organic inner phase into the external aqueous phase^19–22^. The transformation of the emulsion droplets into solid microparticles can be monitored by focused beam reflectance measurement (FBRM) which offers the advantage of in-line measurement of the chord length distribution (CLD) of dispersed particles inside a flowing fluid, without the need of installing a pre-dilution side-stream, as required for other online particle sizing tools. It does not require sampling that could affect the actual particle size distribution due to breakdown or aggregation. FBRM measures a CLD, which is affected by the geometry, size, and number of particles under analysis^23–29^. FBRM will monitor the mechanism and see which parameters can be tweaked to improve drug release or encapsulation performance. The calculation of microparticle formation relies on the reflection of the microparticles and is highly reliant on the particles' optical properties^28–32^. Without the necessity for a pre-dilution side-stream, FBRM allows for in-line estimation of the particle size distribution of scattered particles within a moving fluid. It does not necessitate sampling, which could alter the particle size distribution due to dissolution or aggregation. The FBRM signal is highly dependent on the surface properties of the sample being measured, but it is a useful tool for monitoring the phase^23,28,35–41^. With regard to controlling such a microparticle preparation process the determination of the rate and time point of conversion from liquid droplets into solid particles is of great interest.

Crystallization control, flocculation process design, slurry transfer, polymorphic transition tracking, particle disturbance control, microparticle solidification, protein aggregation and solubility measurements are only a few of the applications that have been identified using FBRM^30,32,42–48^. Despite the fact that several experiments on FBRM have been published in the literature, no online monitoring of the development of microparticles using various polymers has ever been published using FBRM. In light of this, the aim of this study was to examine the ability of FBRM to be used for online monitoring of the shift in the microparticle CLD and detecting the transformation of emulsion droplets into solid microparticles during the solvent evaporation process, as well as to measure the particle size of microparticles with results that are as accurate as those measured by other methods. During the solvent evaporation process, the impact of polymer type on the solidification rate of polymeric microparticles/microparticle blends and particle size/ particle size distribution of emulsion droplets/hardened polymeric microparticles was also investigated. Hardening speed of microparticles, particle size and its distribution of emulsion droplets, and hard microparticles/microparticle blends produced by O/W using different polymers were the parameters examined. Meanwhile, this paper is to study different in-process particle sizing technique (FBRM) and compare it to acknowledged off-line techniques (laser diffraction (LD) and sieve analysis).

## Results and discussion

### Real-time particle size and particle size distribution analyses

FBRM measurement on particle size relatively compared to those obtained using an optical microscope and laser diffraction revealed that there were no variations in particle size of microcapsules (Table 1). To establish this comparison, three batches of microparticles having different sizes were measured with the different equipments to which Focused Beam Reflectance Measurements (FBRM), laser diffraction (LD) and sieve analysis. Particle size analysis using microscopic image and sieve analysis were conducted on final microparticle after the process.

**Table 1:** Effect of polymer type on particle size mean of microparticles.

Polymer	Particle size mean (µm) (± SD)		
	FBRM	Optical microscope	Laser diffraction
Ethyl cellulose 4 cp	83.24 (± 5.28)	88.78 (± 7.64)	84.65 (± 6.11)
Eudragit RL 100	73.42 (± 6.44)	79.62 (± 9.17)	75.37 (± 5.75)
Eudragit RS 100	59.36 (± 5.21)	63.96 (± 8.92)	60.14 (± 6.44)
Polycaprolactone (Mw. 10,000)	51.29 (± 4.09)	57.86 (± 8.12)	53.46 (± 5.32)
PLGA (RG503H)	64.08 (± 3.18)	68.15 (± 6.95)	65.75 (± 4.37)

The results showed that the final particle size measured did not vary as compared to the data collected by optical microscope and laser diffraction measurement (Table 1). It can be concluded that particle size measurement using the centered beam reflectance measurement (FBRM) method yields the same results with particle size data and population patterns of particles in suspension from conventional analysis despite its ability in real time and on-line measurement. The FBRM technique allows for the measurement of parameter processes during particle forming without destroying the morphology or counts of the process's formed particles.

Polymeric microparticles had a diameter ranging from 51 to 83 µm (FBRM) (Table 1). The mean particle size of microparticles made with a high viscosity polymer solution was greater than those made with a low viscosity solution. This is due to faster solidification on the surface of embryonic microparticle droplets, resulting in accelerated microparticle droplet shrinkage. Based on the data, particle size mean which were measured as the square weighted mean chord lengths determined by FBRM were better estimated than those calculated by microscopic observation, as shown by the data's lower standard deviation (Table 1).

Several authors have presented the relationship between particle size distribution (PSD) and chord length distributions (CLD) since the particle chord length is not identical to the generally used particle size. The easiest way to convert a CLD into its corresponding PSD is by developing a PSD–CLD model to calculate CLD corresponding to a known PSD and shape and afterward invert it to obtain a PSD from the CLD (CLD–PSD model)^49^. In 2001, Langston and Jones presented a method in which for a certain PSD of non-spherical particles, the chord length probability distribution is determined by simulating random cuts in the particles^50^. This method is highly dependent on assumptions made during the calculation, and the resulting data are not accurate. Furthermore, FBRM can be applied to track the change in CLD at different levels of microparticle ripening in real time. It can track the shape of microparticles, particle size changes, hardening rate, particle properties, and chord length distribution. The conversion of a PSD from a CLD is an inversion problem, and the most utilized methods to solve this problem include the Least Squares and Constrained Least Squares algorithms^51–56^. For most processes, however, a good precision is often more important than accuracy as the interest relies on the monitoring of process dynamic changes such as particle shape and/or concentration of the suspensions^49,50^.

In microparticulate systems, the solidification rate of polymeric microparticles is a significant parameter that influences particle size, encapsulation strength, and the initial burst^17^. Diffusion of the drug material out of the droplets and precipitation in the exterior phase will result from a very slow hardening of the emulsion droplets. The solubility of the polymer in organic solvents influences the hardening speed of microparticles during the solvent evaporation process, which impacts on microparticle properties including particle size, amount of encapsulated drugs, matrix porosity, solvent residues, and initial drug release^3^. Microparticles have been created using a variety of polymers with various physical properties (such as solubility, molecular weight, reactivity, viscosity, biodegradability, permeability, and so on). Poly(-caprolactone), poly(lactic-co-glycolic acid), Eudragit RS 100, Eudragit RL 100, and ethyl cellulose microparticles are only a few of the materials accessible^4–10^. The microparticle hardening rate of these polymers is unknown. As a result, it's important to understand how polymer properties affect the hardening time of polymeric microparticles.

**Figure 1:**
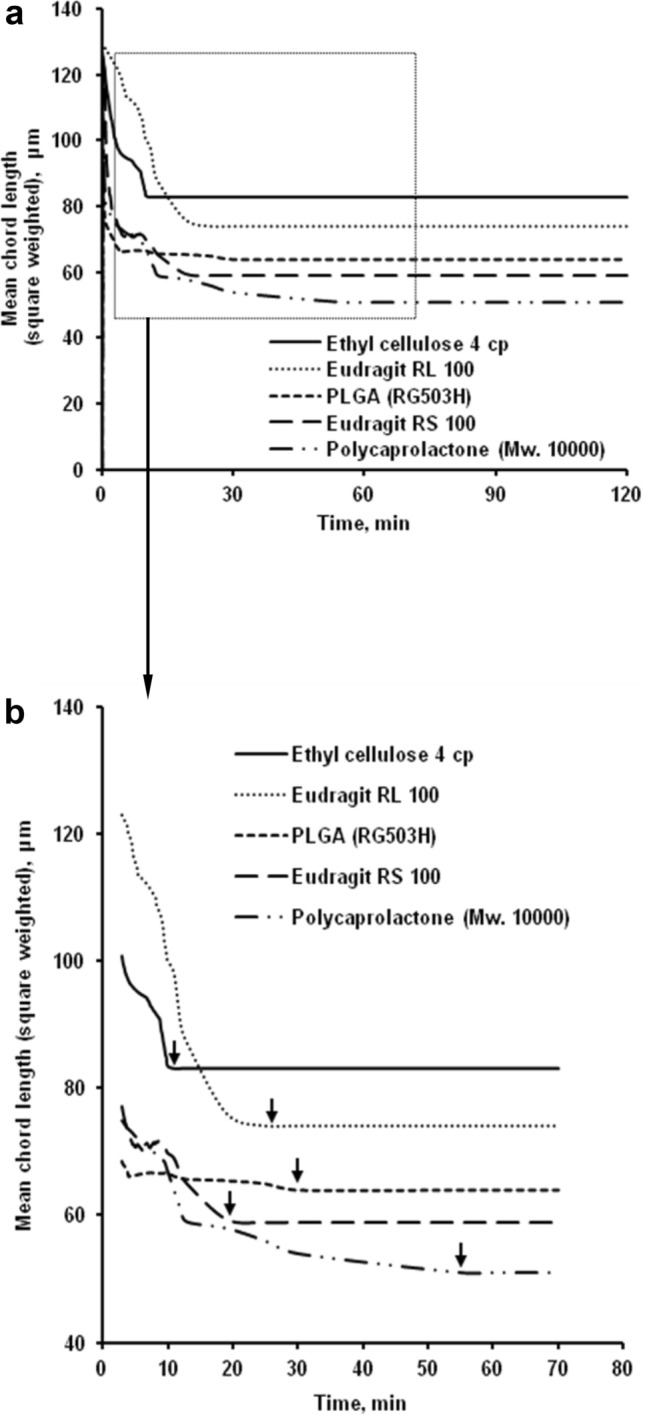
Effect of polymer type on square weighted mean chord length during microparticle formation by O/W method (**a**) whole process and (**b**) hardening time of microparticle; arrow (↓): starting time of microparticle hardening.

The solubility of the polymer in the solvent affected the solidification rate of polymeric microparticles. The solubilities of polymers in dichloromethane were contrasted in this study (Table 2). In dichloromethane, ethyl cellulose (EC) had the lowest solubility, while polycaprolactone had the highest. As a result, polycaprolactone-based microparticles hardened at a slower rate than the others. Due to its poor solubility, ethyl cellulose has the highest hardening rate. In dichloromethane, Eudragit RL 100, Eudragit RS 100, and PLGA (RG503H) is more soluble than ethyl cellulose^5–8,32,57–59^. These properties cause microparticles to solidify at a slower rate than ethyl cellulose. The solidification of polymers with high solubilities took longer time. They remained in the semi-solid state for longer, and it scattered more densely before fully solidifying, resulting in denser microparticles. In dichloromethane, Eudragit RL 100, Eudragit RS 100, and PLGA (RG503H) is more soluble than ethyl cellulose. These properties cause microparticles to solidify at a slower rate than ethyl cellulose. The solidification of polymers with high solubilities took longer^3^. They remained in the semi-solid state for longer, and it scattered more densely before fully solidifying, resulting in denser microparticles^3,6,8^. During solvent evaporation, various types of polymers applied in the formulation of polymeric microparticles result in different particle size, which observed as square weighted mean. FBRM initially observed significant droplet sizes of all types of polymers when the organic polymer solution was emulsified. As a function of time, the square weighted mean chord length of polymeric microparticles is plotted (Fig. 1a). All of the polymers formed small particles (less than 300 µm). On both types of polymers, increasing the process time culminated in a decrease in particle size followed by a plateau size where the particle size remained unchanged. The particle size mean of microparticles when ethyl cellulose was utilized was greater than the others. The viscosity of the polymer solution has an effect on it. The high viscosity of Ethyl cellulose emulsion droplets reduced the organic phase's dispersibility in the aqueous media, resulting in larger particles.

**Table 2:** Effect of polymer type on solubility in dichloromethane, viscosity of polymeric solution and particle size mean of microparticles.

Polymer	Solubility (g/ml) (± SD)	Viscosity (cSt) (± SD)	Hardening time (min)
Ethyl cellulose 4 cp	0.86 (± 0.03)	10.31 (± 1.14)	10.5
Eudragit RL 100	1.04 (± 0.04)	4.72 (± 0.83)	25
Eudragit RS 100	1.42 (± 0.02)	3.84 (± 0.39)	19
Polycaprolactone (Mw. 10,000)	1.89 (± 0.05)	3.15 (± 0.45)	55
PLGA (RG503H)	1.25 (± 0.06)	4.36 (± 0.52)	30

For both polymers, the droplet shrinkage may be divided into two phases. Within 9 min (EC), 15 min (Eudragit RS 100), 20 min (Eudragit RL 100), and 25 min (PLGA), the original droplet size shrank dramatically (RG503H). This was accompanied by a period with no further pronounced shrinkage, known as a discontinued or sluggish shrinkage phase (Fig. 1a). It shows that in the first (rapid) step, the solvent was rapidly removed, and that in the second (slow) phase, the embryonic microparticle droplets were transformed into stable microparticles. Polycaprolactone caused the droplet size decrease to occur for another 55 min, meaning that the embryonic microparticle droplets solidified between 50 and 55 min (Fig. 1a). The start of the plateau process for all polymers was 10.5 min (ethyl cellulose 4 cp), 25 min (Eudragit RL 100), 19 min (Eudragit RS 100), 55 min (polycaprolactone), and 30 min (PLGA (RG503H) based on FBRM results (Fig. 1b and Table 2).

As EC microparticles are extracted quickly with solvent (Fig. 1), the polymer solidifies quickly on the droplet surface^21,37^, resulting in diffuse scattering to some extent. The improvement in the FBRM signal must be interpreted as opacification shifts and particle solidification.

**Figure 2:**
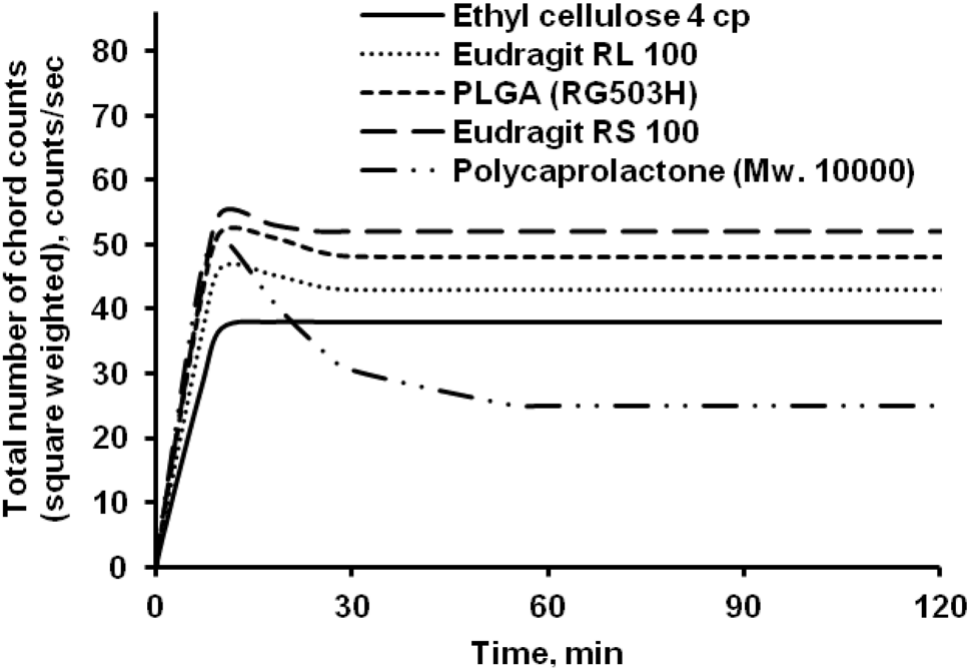
Effect of polymer type on the number of chord counts (square weighted) during solvent evaporation process.

As a time function, the chord count or particle counts of polymeric microparticles reveal a curve (Fig. 2). FBRM initially observed lower chord counts as the organic polymer solution was emulsified; but, as the process time was increased, the chord counts increased, indicating an increase in particle counts, followed by a plateau period where the chord counts were stable. During the solvent evaporation process, Eudragit RL 100, PLGA (RG503H), Eudragit RS 100, and polycaprolactone showed identical chord counts profiles. The particle counts of ethyl cellulose microparticles is smaller than those of the other microparticles, owing to the processing of microparticles of the biggest size.

Eudragit RL 100, Eudragit RS 100, and PLGA (RG503H) had smaller particle sizes than ethyl cellulose, resulting in higher particle counts. The particle size of polycaprolactone was the lowest, but the particle count was not the largest. This is due to Polycaprolactone's particle properties, which create slightly transparent microparticles as opposed to other polymers.

**Figure 3:**
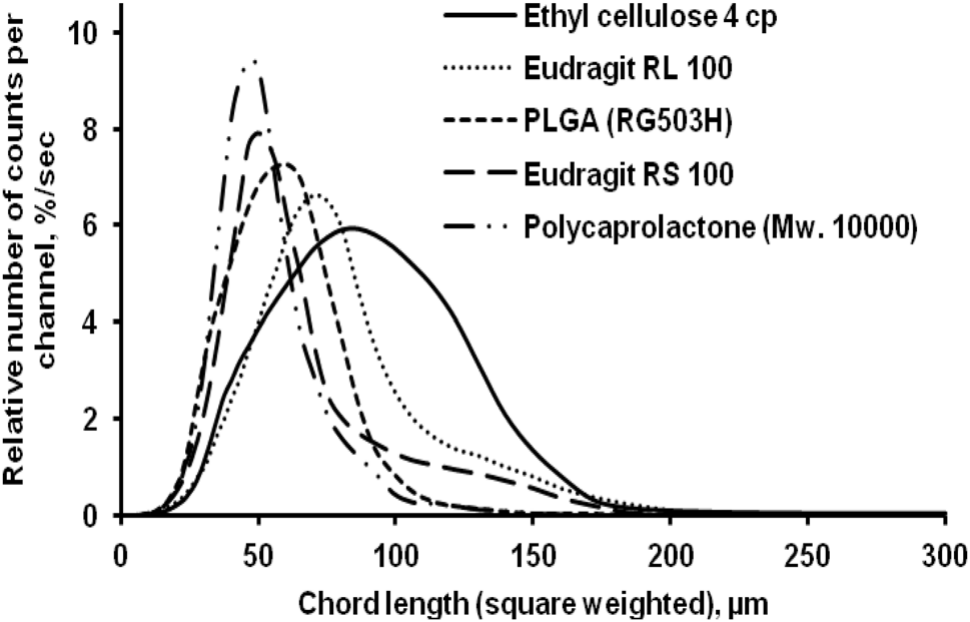
Effect of polymer type on the square weighted chord length distributions, (particle size distribution) at 4 h stirring time.

The formation of microparticles-based polymer materials was clearly supported by real-time particle size analysis and particle size distribution experiments of all polymeric microparticles with in situ polymerization. Surprisingly, these findings suggest that, based on the polymer structures, all of the polymer products used various microparticle forming mechanisms. At the same concentration, the chord length distributions (particle size distribution) of various polymers determined by FBRM is different (Fig. 3). Since the number of microparticles was reduced, larger microparticles resulted in longer chord lengths and a lower peak particle number. In this case, increasing the viscosity of the polymer solution resulted in a larger square weighted mean chord length (particle size) and a wider chord length distribution. Polycaprolactone solution (organic phase) had a lower viscosity than other polymers, resulting in smaller microparticles. In comparison to other polymeric microparticles, this polymer provided narrower square weighted mean chord lengths and narrow chord length distributions.

**Figure 4:**
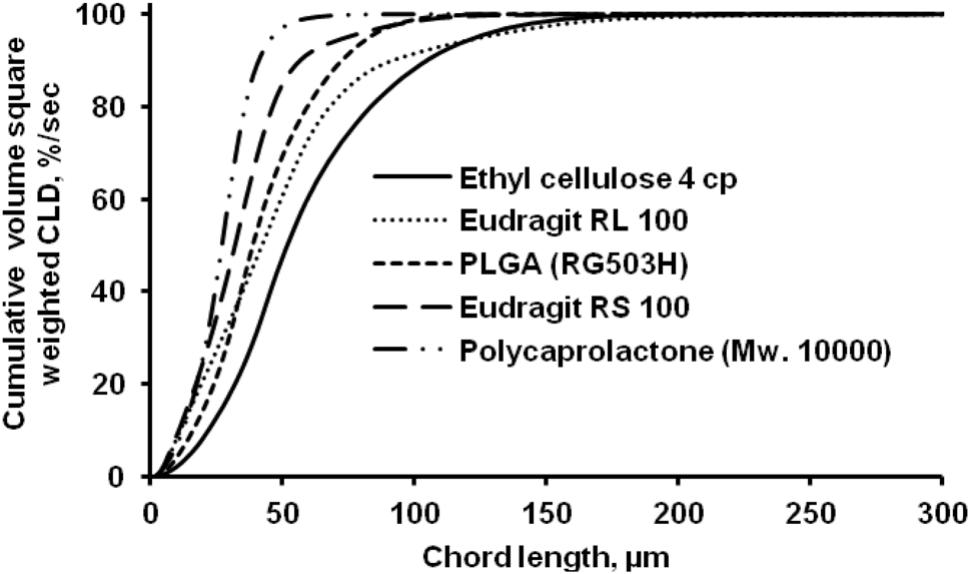
Comparison of the square weighted chord length distribution for various polymer obtained by the FBRM method (O/W) at 4 h stirring time.

Figure 4 shows the total volume square weighted chord length distributions of polymeric microparticles made by the O/W process. Polymeric microparticles with a smaller square weighted mean chord length would possess more fine and intermediate microparticles in their scale fraction.

### Scanning electron microscopy (SEM) studies

**Figure 5:**
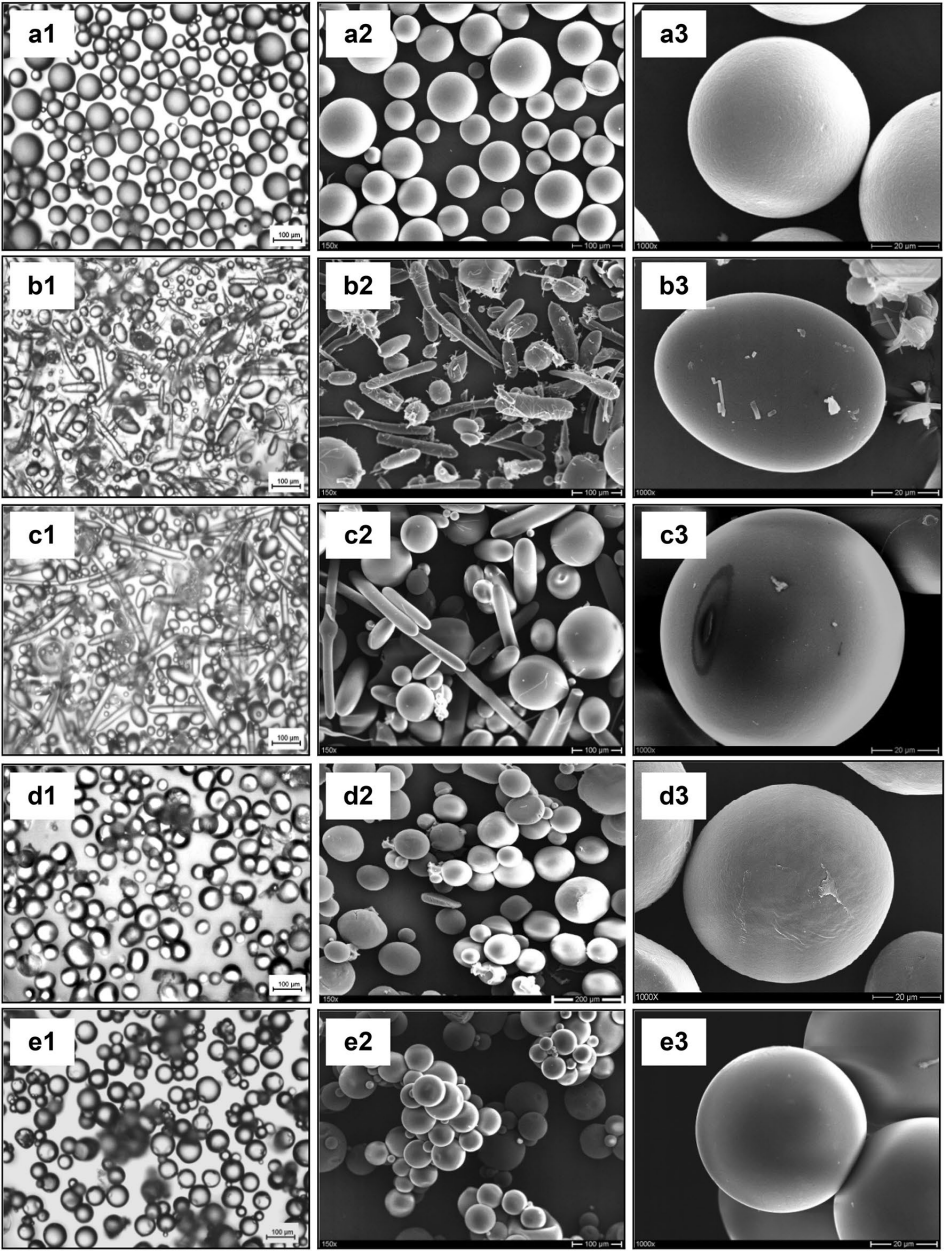
Optical microscopy pictures (1) and SEM pictures of polymeric microparticles (2. at 150 × magnification & 3. at 1000 × magnification) (**a**) Ethyl cellulose 4 cp; (**b**) Eudragit RL 100; (**c**) Eudragit RS 100; (**d**) Polycaprolactone (Mw. 10,000); (**e**) PLGA (RG503H).

Figure 5 shows Optical microscopy pictures (Fig. 5a1–e1) and SEM photomicrograph of microparticles produced by the O/W process with the polymers ethyl cellulose (EC), Eudragit RL 100, Eudragit RS 100, polycaprolactone, and PLGA (RG503H) (Fig. 5a2–e2 and a3–e3). Because of the influence of the form and physical properties of the polymer, as well as its solubility in dichloromethane as a solvent, all microparticles possess varying levels of opacity (Fig. 5a1–e1). Polymers with a high dichloromethane solubility took longer time to solidify and remained in a semisolid state. When the polymer matrix was shrunk for a longer period of time, the polymer matrix becomes thick, and the droplet gradually shrank into stable microparticles, leaving transparent droplets. When a laser beam strikes a transparent microparticle, multiple reflections occur inside the microparticle, illuminating the whole sphere. As a more opaque microparticle is struck by a laser pulse, the light is dispersed to the detector, resulting in a higher scattering value^36,37,41,60^. The backscattered signal is very strong due to the invisible microparticles' absorbance, resulting in a high degree of chord length (particle size) and low chord counts (particle counts)^61–63^. This is in conjunction with Greaves et al., as well as Sparks and Dobbs, who concluded that only opaque and highly reflective droplets or microparticles (with microstructure on the surface) provide repeatable and consistent effects^41,61,62^.

Microparticles of ethyl cellulose (EC), polycaprolactone, and PLGA (RG503H) were spherical with smooth surfaces without aggregation, while those of Eudragit RL 100 and Eudragit RS 100 were spherical, oval, and needle shaped (mixture) with smooth surfaces without aggregation, according to the surface of microparticles without drug prepared by the O/W process. There were no pores on any of the microparticle surfaces (Fig. 5a3–e3).

## Conclusion

For all three processed batches analyzed, FBRM, laser diffraction, and sieve analysis revealed identical particle sizes. The FBRM approach has the advantage of being able to do particle size analysis in real time without sampling or dilution. During the solvent evaporation process, the FBRM was successfully used as an efficient process to analyze either quantitative particle size estimation or qualitative online monitoring of the change in the microparticle particle size distribution. The FBRM detects the transition of emulsion droplets into solid microparticles and agglomeration by detecting a shift in signal that is caused by particle surface characteristics and its optical properties.

## Experimental section

### Materials

All materials were of at least reagent grade and used as received: Ethocel (Standard 4 Premium, Colorcon Ltd, Kent, UK)); ethyl acrylate methyl methacrylate copolymer (Eudragit RS 100 and Eudragit RL 100, Evonik Röhm GmbH, Darmstadt, Germany), Poly(D,L-lactide-co-glycolide) (Resomer RG503H, Boehringer-Ingelheim Pharma GmbH & Co. KG, Ingelheim, Germany), Poly(ε-caprolactone) (PCL; Mn approx. 10,000) (Sigma-Aldrich Chemie GmbH, Steinheim, Germany); polyvinyl alcohol (PVA, Mowiol 40–88, Kuraray Europe GmbH, Frankfurt, Germany); and dichloromethane (Carl Roth GmbH & Co. KG, Karlsruhe, Germany).

### Viscosity measurement

7.5% w/v solution of Ethocel 4 cP, Eudragit RS 100, Eudragit RL 100, PLGA (Resomer RG503H) and poly(ε-caprolactone) in dichloromethane were analyzed using an Ostwald viscometer type 50,111/Ia, instrument constant: K = 0.05152 mm^2^/s^2^ (Schott-Geräte GmbH, Hofheim, Germany) at 25 °C (n = 3). The viscosity were calculated as follows:

ν = K.t.

ν : kinematic viscosity (mm^2^/s or cSt).

K : instrument constant (mm^2^/s^2^).

t : flow time (s).

### Microparticles preparation

The solvent evaporation method based on the formation of O/W emulsion was used to prepare microparticles. In the O/W-dispersion method, a solution of the polymer in dichloromethane (7.5% w/v) was dispersed into an external aqueous phase (800 ml 0.25% PVA solution). The emulsion was stirred for 4 h at 500 rpm with a propeller stirrer (Heidolph Elektro GmbH & Co. KG, Kelheim, Germany). After 4 h, the microparticles were separated from the external aqueous phase by wet sieving followed by washing with 200 ml deionized water, desiccator-drying for 24 h and storage in a desiccator.

### Comparison of average particle size by various techniques

#### Focused beam reflectance measurements (FBRM)

**Figure 6:**
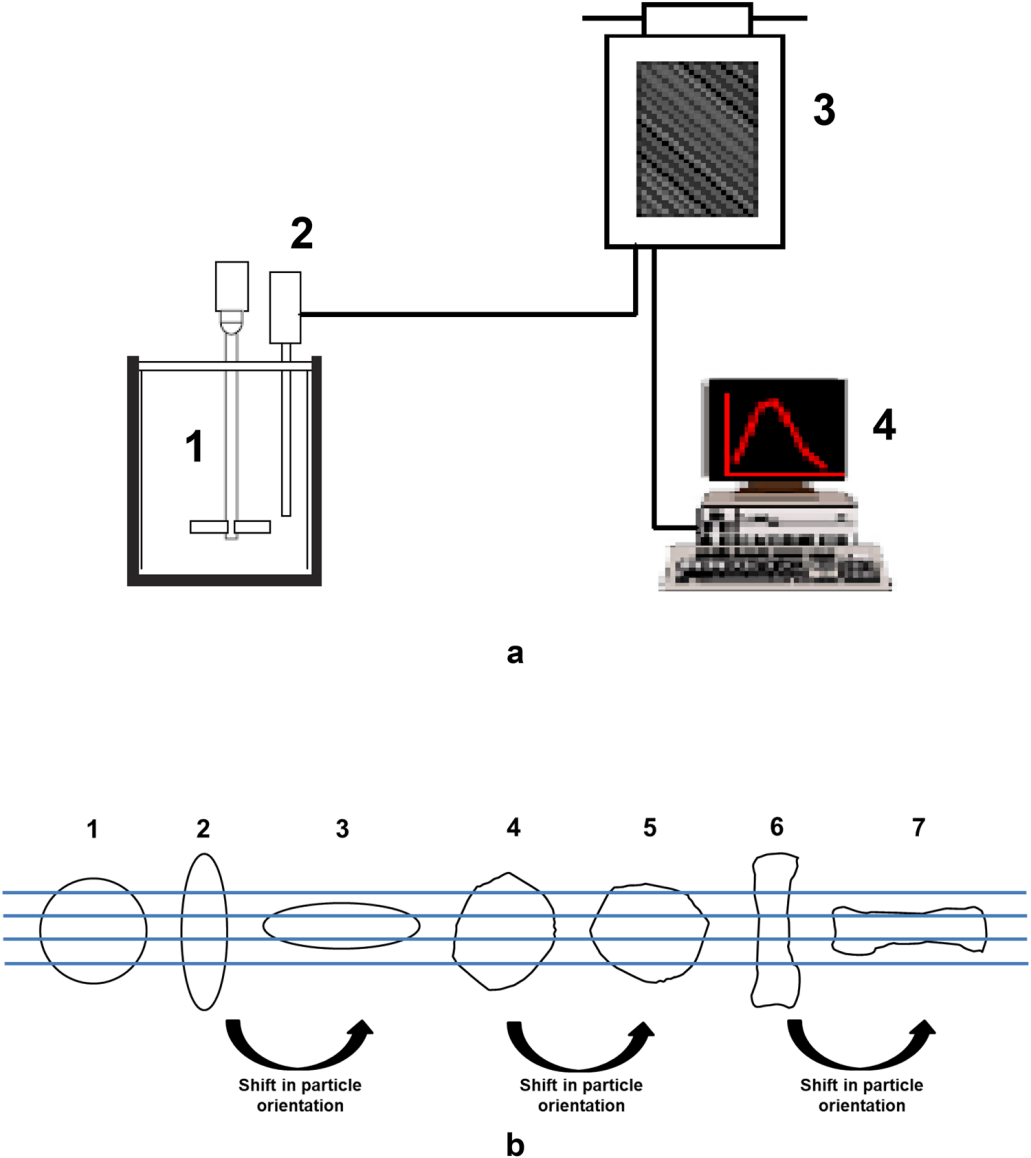
Schematic drawing of probe positioning relative to the impeller (1. Propeller stirrer; 2. FBRM probe; 3. Processing unit; 4. PC monitoring the particle size distribution on-line) (**a**), and Examples of the measured chord length (line) when a laser beam crosses (1) a spherical particle, (2 and 3) an oval particle in different positions and (4, 5, 6 and 7) an irregular particle in different positions—illustration of the effect of particle orientation on the obtained chord length (**b**).

FBRM probe (FBRM D600T, Mettler Toledo AutoChem, Inc., Redmond, WA, USA) was immersed and positioned in the emulsification vessel (O/W emulsions). It was placed between the propeller stirrer and inner side of the emulsion vessel (Fig. 6). This spot can provide good flow of turbulence, hence allowing a representative sample of the particle system to be measured. The measurement range of the FBRM D600T probe is 0.25—4000 μm. A propeller stirrer (Heidolph Elektro GmbH & Co. KG, Kelheim, Germany) was set at stirring speed of 500 rpm for 4 h. The measurements were performed in triplicate every 10 s, during a period of 4 h. The FBRM D600T use a beam of laser light which rotated with constant speed of 2 ms^−1^as source. The of laser energy is reflected back into the probe by backscatter from particles next to the sapphire window as an orifice. Figure 6 shows the operating principle of the FBRM probe^26,30,63^.

A detailed operating mechanism of the FBRM technique is described by Kougoulos et al.^30^ The measured particle size when a laser beam crosses the particle randomly for spherical, irregular and odd-shaped particles, shape and orientation will influence the measured particle size is described by Silva et al.^63^ The operating mechanism of the FBRM technique^30^ and the measuring particle size when a laser beam crosses the particle randomly^63^ is acknowledged.

#### Laser diffraction

The Mastersizer 2000 laser diffraction instrument with the Hydro 2000S liquid cell (Malvern Instruments Inc., MA, USA) was used to perform the particle size analysis of the polymeric microparticles. During a measurement, scattered light intensity at various scattering angles was first collected, which was then deconvoluted to a particle size distribution using a vendor proprietary algorithm based on the Fraunhofer approximation^33,63^. The solid concentration was monitored and controlled by the % obscuration. The obscuration is defined as the percentage of intensity loss of incident light after passing through a scattering medium, in this case, the polymeric microparticles suspension. The % obscuration is proportional to solid concentration in diluted region where no multiple light scattering is present.

The measurement principle of FBRM differs fundamentally from other established particle sizing methods. It is based on determining the time length of a backscattered light impulse which occurs when the spot of a moving laser beam crosses a particle. Because the velocity of the laser spot is constant the time span of backscattering is directly proportional to the path length traversed by the spot on the particles projection area. However, as the laser spot crosses a particle not necessarily at its centre but randomly between two opposite edges, even from monodisperse particles a broad distribution of different chord lengths is obtained rather than a discrete diameter. Using an idealised two- dimensional model, the chord length distribution (CLD) of sphe- rical particles can be calculated according to Eq. (1) (Simmons et al., 1999; Tadayyon and Rohani, 1998).

#### Sieve analysis

Sieve analysis was performed, in triplicate, on 20 g of sample from each batch. Nine sieves with mesh sizes of 2000, 1400, 1000, 500, 315, 250, 180, 100, and 50 μm were stacked. A collector pan was placed below the sieve with the smallest mesh size. The samples were placed on the top sieve (2000 μm) and a lid was placed on it. The assembly was vibrated on an automatic sieve shaker (VE 1000, Retsch, Haan, Germany) for 5 min with an amplitude of 2 mm. Such gentle conditions were chosen to prevent breakage of the polymeric micropaticles samples. After shaking, each sieve was weighted individually and the mass percentage of material retained on each sieve was calculated.

### Characterization techniques

#### Optical microscopy

Microparticles were spread on microscope slides and observed with an optical light microscope (Axiotrop 50, Carl Zeiss AG, Jena, Germany) equipped with an image analysis system (INTEQ Informationstechnik GmbH, Berlin, Germany) consisting of a digital camera (type MC1) and the software for measuring particles (version 1.4.1). Micropaticles samples after sieving process were spread individually in water, the data of particle size from sample were collected by manual calculation in macroscope connected into camera.

#### Scanning electron microscopic studies

The external morphology of microparticles was analysed by scanning electron microscopy (SEM). For surface imaging, the microparticles were fixed on a sample holder with double-sided tape. All samples were coated under an argon atmosphere with fine gold to a thickness of 8 nm (SCD 040, Bal-Tec GmbH, Witten, Germany) in a high-vacuum evaporator. Samples were then observed with a scanning electron microscope (S-4000, Hitachi High-Technologies Europe GmbH, Krefeld, Germany).

## References

[1] Alhnan MA, Basit AW (2011). Engineering polymer blend microparticles: An investigation into the influence of polymer blend distribution and interaction. Eur. J. Pharm. Sci..

[2] Saralidze K, Koole LH, Knetsch MLW (2010). Polymeric microspheres for medical applications. Materials.

[3] Spörl JM, Batti F, Vocht MP, Raab R, Müller A, Hermanutz F, Buchmeiser MR (2018). Ionic liquid approach toward manufacture and full recycling of all-cellulose composites. Macromol. Mater. Eng..

[4] Chen DR, Bei JZ, Wang SG (2000). Polycaprolactone microparticles and their biodegradation. Polym. Degrad. Stabil..

[5] Gibaud S, Awwadi NJA, Ducki C, Astier A (2004). Poly(ε-caprolactone) and Eudragit^®^ microparticles containing fludrocortisone acetate. Int. J. Pharm..

[6] Chen D, Chen H, Bei J, Wang S (2000). Morphology and biodegradation of microspheres of polyester-polyether block copolymer based on polycaprolactone/polylactide/poly(ethylene oxide*)*. Polym. Int..

[7] Jeyanthi R, Metha RC, Thanoo BC, Deluca PP (1997). Effect of processing parameters on the properties of peptide containing PLGA microspheres. J. Microencap..

[8] Li WI, Anderson KW, Mehta RC, DeLuca PP (1995). Prediction of solvent removal profile and effect on properties for peptide-loaded PLGA microspheres prepared by solvent extraction/evaporation method. J. Control. Rel..

[9] Murtaza G (2012). Ethylcellulose microparticles: A review. Drug Res..

[10] Sinha VR, Bansal K, Kaushik R, Kumria R, Trehan A (2004). Poly-β-caprolactone microspheres and nanospheres: An overview. Inter. J. Pharm..

[11] Souza MC, Marchetti JM (2011). Development of albendazole sulfoxide-loaded Eudragit microparticles: A potential strategy to improve the drug bioavailability. Adv. Powder Technol..

[12] Trapani A, Laquintana V, Denora N, Lopedota A, Cutrignelli A, Franco M, Trapani G, Liso G (2007). Eudragit RS 100 microparticles containing 2-hydroxypropyl-β-cyclodextrin and glutathione: Physicochemical characterization, drug release and transport studies. Eur. J. Pharm. Sci..

[13] Mehta RC, Thanoo BC, DeLuca PP (1996). Peptide containing microspheres from low molecular weight and hydrophilic poly(D, L-lactide-co-glycolide). J. Control. Rel..

[14] Li H, Ma Y, Li Z, Cui Y, Wang H (2018). Synthesis of novel multilayer composite microcapsules and their application in self-lubricating polymer composites. Compos. Sci. Technol..

[15] Ray AML, Chiffoleau S, Iooss P, Grimandi G, Gouyette A, Daculsi G, Merle C (2003). Vancomycin encapsulation in biodegradable poly(ε-caprolactone) microparticles for bone implantation Influence of the formulation process on size, drug loading, in vitro release and cytocompatibility. Biomaterials.

[16] Duarte ARC, Gordillo MD, Cardoso MM, Simplicio AL, Duarte CMM (2006). Preparation of ethyl cellulose/methyl cellulose blends by supercritical antisolvent precipitation. Int. J. Pharm..

[17] Muhaimin M, Bodmeier R (2020). Data on the application of the focused beam reflectance measurement (FBRM): A process parameters dataset for the ethyl cellulose (EC) microparticles preparation by the solvent evaporation method. Data Brief.

[18] Pearnchob N, Bodmeier R (2003). Dry polymer powder coating and comparison with conventional liquid-based coatings for Eudragit^®^ RS, ethylcellulose and shellac. Eur. J. Pharm. Biopharm..

[19] Muhaimin, M., Yusnaidar, Y., Syahri, W., Latief, M., Chaerunisaa, A.Y. Microencapsulation of *Macaranga gigantea* leaf extracts: Production and Characterization. *Pharmacognosy J.* 12(4), (2020).

[20] Saravanan M, Anupama B (2011). Development and evaluation of ethylcellulose floating microspheres loaded with ranitidine hydrochloride by novel solvent evaporation-matrix erosion method. Carbohyd. Polym..

[21] Freitas S, Merkle HP, Gander B (2005). Microencapsulation by solvent extraction/evaporation: reviewing the state of the art of microsphere preparation process technology. J. Control. Rel..

[22] O’Donell PB, McGinity JW (1999). Preparation of microspheres by the solvent evaporation technique. Adv. Drug Delivery Rev..

[23] Boxall JA, Koh CA, Sloan ED, Sum AK, Wu DT (2010). Measurement and calibration of droplet size distributions in water-in-oil emulsions by particle video microscope and a focused beam reflectance method. Ind. Eng. Chem. Res..

[24] Pandit A, Katkar V, Ranade V, Bhambure R (2019). Real-time monitoring of biopharmaceutical crystallization: chord length distribution to crystal size distribution for lysozyme, rhu insulin, and vitamin B12. Ind. Eng. Chem. Res..

[25] Acevedo, D., Wu, W. , Yang, X., Pavurala, N., Mohammad, A., O'Connor*,* T. Evaluation of focused beam reflectance measurement (FBRM) for monitoring and predicting crystal size of carbamazepine in crystallization processes. *Cryst. Eng. Commun*. 2020.

[26] Dowding PJ, Goodwin JW, Vincent B (2001). Factors governing emulsion droplet and solid particle size measurements performed using the focused beam reflectance technique. Colloids Surf. A: Physicochem. Eng. Asp..

[27] Heath AR, Fawell PD, Bahri PA, Swift JD (2002). Estimating average particle size by focused beam reflectance measurement (FBRM). Part. Part. Syst. Char..

[28] Kail N, Marquardt W, Briesen H (2009). Estimation of particle size distributions from focused beam reflectance measurements based on an optical model. Chem. Eng. Sci..

[29] Kirwan LJ (2009). Investigating bauxite residue flocculation by hydroxamate and polyacrylate flocculants utilising the focussed beam reflectance measurement probe. Int. J. Miner. Process..

[30] Kougoulos E, Jones AG, Kaczmar MW (2005). Modelling particle disruption of an organic fine chemical compound using Lasentec focussed beam reflectance monitoring (FBRM) in agitated suspensions. Powder Technol..

[31] Leba H, Cameirao A, Herri JM, Darbouret M, Peytavy JL (2010). Chord length distributions measurements during crystallization and agglomeration of gas hydrate in a water-in-oil emulsion: Simulation and experimentation. Chem. Eng. Sci..

[32] Muhaimin M, Bodmeier R (2017). Effect of solvent type on preparation of ethyl cellulose microparticles by solvent evaporation method with double emulsion system using focused beam reflectance measurement. Polymer Int..

[33] Ma Z, Merkus HG, de Smet JGAE, Heffels C, Scarlett B (2000). New developments in particle characterization by laser diffraction: Size and shape. Powder Technol..

[34] Rawle A (1993). Basic Principles of Particle Size Analysis.

[35] Wynn EJW (2003). Relationship between particle-size and chord-length distributions in focused beam reflectance measurement: Stability of direct inversion and weighting. Powder Technol..

[36] Vay K, Frieβ W, Scheler S (2012). Understanding reflection behavior as a key for interpreting complex signals in FBRM monitoring of microparticle preparation processes. Int. J. Pharm..

[37] Yu W, Erickson K (2008). Chord length characterization using focused beam reflectance measurement probe—Methodologies and pitfalls. Powder Technol..

[38] Zidan AS, Rahman Z, Khan MA (2010). Online monitoring of PLGA microparticles formation using Lasentec focused beam reflectance (FBRM) and particle video microscope (PVM). AAPS J..

[39] Kempkes M, Eggers J, Mazzotti M (2008). Measurement of particle size and shape by FBRM and in situ microscopy. Chem. Eng. Sci..

[40] Simmons MJH, Langston PA, Burbidge AS (1999). Particle and droplet size analysis from chord distributions. Powder Technol..

[41] Greaves D, Boxall J, Mulligan J, Montesi A, Creek J, Sloan ED, Koh CA (2008). Measuring the particle size of a known distribution using the focused beam reflectance measurement technique. Chem. Eng. Sci..

[42] Abbas A, Nobbs D, Romagnoli JA (2002). Investigation of on-line optical particle characterization in reaction and cooling crystallization systems, Current state of the art. Measure. Sci. Technol..

[43] Barrett P, Glennon B (2002). Characterizing the metastable zone width and solubility curve using Lasentec FBRM and PVM. Trans IChemE..

[44] Yu ZQ, Tan RBH, Chow PS (2005). Effects of operating conditions on agglomeration and habit of paracetamol crystals in anti-solvent crystallization. J. Cryst. Growth..

[45] Sankaranarayanan S, Likozar B, Navia R (2019). Real-time particle size analysis using the focused beam reflectance measurement probe for in situ fabrication of polyacrylamide–filler composite materials. Sci. Rep..

[46] Daymo EA, Hylton TD, May TH (1998). Acceptance testing of the lasentec focused beam reflectance measurement (FBRM) monitor for slurry transfer applications at Hanford and Oak Ridge. Part SPIE Conf. Nuclear Waste Instrum. Eng. Boston Massachusetts.

[47] O'Sullivan B, Barrett P, Hsiao G, Carr A, Glennon B (2003). In situ monitoring of polymorphic transitions. Organic Process Res. Develop..

[48] Kim YS, Rio JRMD, Rousseau RW (2005). Solubility and prediction of the heat of solution of sodium naproxen in aqueous solutions. J. Pharm. Sci..

[49] Li MZ, Wilkinson D, Patchigolla K (2005). Determination of non-spherical particle size distribution from chord length measurements. Part 2: Experimental validation. Chem. Eng. Sci..

[50] Langston PA, Jones TF (2001). Non-spherical 2-dimensional particle size analysis from chord measurements using Bayes’ theorem. Part. Part. Syst. Charact..

[51] Bloemen HHJ, De Kroon MGM (2005). Transformation of chord length distributions into particle size distributions using least squares techniques. Part. Sci. Technol..

[52] Langston PA (2002). Comparison of least-squares method and Bayes’ theorem for deconvolution of mixture composition. Chem. Eng. Sci..

[53] Langston PA, Burbidge AS, Jones TF, Simmons MJH (2001). Particle and droplet size analysis from chord measurements using Bayes’ theorem. Powder Technol..

[54] Liu WD, Clark NN, Karamavruc AI (1998). Relationship between bubble size distributions and chord-length distribution in heterogeneously bubbling systems. Chem. Eng. Sci..

[55] Tadayyon A, Rohani S (1998). Determination of particle size distribution by Par-Tec (R) 100: Modeling and experimental results. Part. Part. Syst. Charact..

[56] Ruf A, Worlitschek J, Mazzotti M (2000). Modeling and experimental analysis of PSD measurements through FBRM. Part. Part. Syst. Charact..

[57] Turner DJ, Miller KT, Sloan ED (2009). Direct conversion of water droplets to methane hydrate in crude oil. Chem. Eng. Sci..

[58] Kumar V, Taylor MK, Mehrotra A, Stagner WC (2013). Real-time particle size analysis using focused beam reflectance measurement as a process analytical technology tool for a continuous granulation-drying-milling process. AAPS Pharm. Sci. Tech..

[59] Yeo Y, Park K (2004). Control of encapsulation efficiency and initial burst in polymeric microparticle systems. Arch. Pharmacal Res..

[60] Wu H, White M, Khan MA (2011). Quality-by-Design (QbD): An integrated process analytical technology (PAT) approach for a dynamic pharmaceutical co-precipitation process characterization and process design space development. Int. J. Pharm..

[61] Sparks RG, Dobbs CL (1993). The use of laser backscatter instrumentation for the online measurement of the particle-size distribution of emulsions. Part. Part. Syst. Char..

[62] Scheler S (2013). Ray tracing as a supportive tool for interpretation of FBRM signals from spherical particles. Chem. Eng. Sci..

[63] Silva, A.F.T., Burggraeve, A., Denon, Q., Van der Meeren, P., Sandler, N., Van Den Kerkhof, T., Hellings, M., Vervaet, C., Remon, J.P., Lopes, J.A., De Beer, T. Particle sizing measurements in pharmaceutical applications: Comparison of in-process methods versus off-line methods. *Eur. J. Pharm. Biopharm.* (2013). 10.1016/j.ejpb.2013.03.032 23583493

